# Validation of Skeletal Muscle *cis*-Regulatory Module Predictions Reveals Nucleotide Composition Bias in Functional Enhancers

**DOI:** 10.1371/journal.pcbi.1002256

**Published:** 2011-12-01

**Authors:** Andrew T. Kwon, Alice Yi Chou, David J. Arenillas, Wyeth W. Wasserman

**Affiliations:** Centre for Molecular Medicine and Therapeutics, Child and Family Research Institute, Genetics Graduate Program, and Department of Medical Genetics, University of British Columbia, Vancouver, British Columbia, Canada; University of Oxford, United Kingdom

## Abstract

We performed a genome-wide scan for muscle-specific *cis*-regulatory modules (CRMs) using three computational prediction programs. Based on the predictions, 339 candidate CRMs were tested in cell culture with NIH3T3 fibroblasts and C2C12 myoblasts for capacity to direct selective reporter gene expression to differentiated C2C12 myotubes. A subset of 19 CRMs validated as functional in the assay. The rate of predictive success reveals striking limitations of computational regulatory sequence analysis methods for CRM discovery. Motif-based methods performed no better than predictions based only on sequence conservation. Analysis of the properties of the functional sequences relative to inactive sequences identifies nucleotide sequence composition can be an important characteristic to incorporate in future methods for improved predictive specificity. Muscle-related TFBSs predicted within the functional sequences display greater sequence conservation than non-TFBS flanking regions. Comparison with recent MyoD and histone modification ChIP-Seq data supports the validity of the functional regions.

## Author Summary

For efficient identification of genomic sequences responsible for regulating gene expression, a number of computer programs have been developed for automatic annotation of these regulatory regions. We searched for potential regulatory regions responsible for controlling the expression of skeletal muscle-specific genes using these programs, and validated the predictions in a popular cell culture model for muscle. We were able to identify 19 previously uncharacterized regulatory regions for muscle genes. The accuracy of the predictions made by these programs leaves much to be desired, leading us to conclude that other signals in addition to the sequence information will be required to achieve sufficient predictive power for genome annotation. Genomic regions with confirmed regulatory function were compared against non-functional sequences, revealing sequence conservation, composition and chromatin modification properties as important signals in determining regulatory region functionality.

## Introduction

A regulatory network represents the complex interplay between regulatory proteins and biochemical processes that govern when and where genes are expressed. Two important components of a regulatory network are *cis*-regulatory modules (CRM), composed of functionally interacting clusters of transcription factor binding sites (TFBS) sufficient to confer a pattern of expression upon a promoter, and the corresponding trans-acting transcription factors (TFs) that bind to a CRM to regulate transcription initiation. The multiple TFBS that constitute a CRM allow for combinatorial control of expression; a limited number of TFs can participate in an exponential number of combinations with each potentially conferring specific patterns of gene activity [1].

CRMs can be situated almost anywhere relative to the structure of a gene: both near and far (even exceptionally far) from the promoter region(s) at which transcription initiates. While there are indications of quantitative orientation effects in some cases [2], CRMs are generally thought to be active in either direction relative to a gene promoter. Linear distance in primary sequence is no indication of the three dimensional distance (or orientation) within the nucleus. Regulatory regions can be located in introns of an adjacent gene [3], [4], can skip over intervening genes [5] and there are suggestions that CRMs can act on genes located on different chromosomes [3], [6]. Reflecting these properties, the discovery of CRMs stands out as a significant challenge for both computational and experimental research.

In multicellular organisms, maintaining precise spatial and temporal control of transcription in various cell types is vital for correct tissue development and specialization [7]–[9]. One of the most widely studied “programs” of tissue development is the regulation of skeletal muscle differentiation. Myogenesis is a structured process, in which mononucleate myoblasts fuse together to form multinucleate myotubes, which then develop into classes of myofibres [10]. C2C12 cells provide a popular model for this process, with an easily triggered switch between the growth and differentiation phases [11]. Any tissue differentiation process requires complex transcriptional regulation controls. For skeletal muscle, differentiated cell gene expression involves at least two major TF families, the myogenin family and the MADS family [12]–[14]. In many differentiation processes, multiple proteins within a homology-based family can participate in the regulatory control of gene expression at overlapping temporal stages of the process. Skeletal muscle differentiation follows this model; thus the myogenin family may equally refer to Myogenin, MyoD, and Myf-2 while the MADS set encompasses both Srf and multiple members of the Mef2 gene family. Dozens of muscle-specific CRMs have been identified [15]–[17], usually based on reporter gene assays in the C2C12 cell culture myogenesis model.

Aided by the relatively plentiful set of skeletal muscle CRMs, much effort has been made by the bioinformatics research community to develop predictive algorithms for CRM discrimination. Multiple CRM detection programs have been developed, which look for clusters of TFBS specific to the TFs known to be involved in the cell type of interest. An original discriminative method to distinguish between CRM and non-CRM sequences based on a logistic regression analysis (LRA) procedure has been followed by a plethora of more advanced approaches (Supplemental Table S1 in Text S1) [15]. For example, MSCAN makes use of motif-specific p-values to compute the statistical significance of sets of non-overlapping potential TFBSs [18], while ClusterBuster is based on a hidden Markov model that incorporates heuristics to improve predictive performance [19]. None of the methods are sufficiently reliable for direct genome annotation; the specificity of predictions is sufficiently low that laboratory validation is essential to distinguish functional CRMs. The overall performance of the methods and the properties that differentiate the functional CRMs from the false candidates remain to be determined.

In some cases, the prediction of CRMs has been coupled with phylogenetic footprinting under the premise that sequence conservation of known CRMs and TFBS is indicative of function and therefore a conservation filter will improve the positive predictive value of the CRM prediction methods [15], [20]–[22]. It is often the case that the regulatory sequences display evidence of evolutionary selective pressure compared to the background rates of sequence change in non-functional sequence [23], [24]. If the expression pattern of a gene is conserved between two species in the same taxonomy class, the CRM that confers the pattern is likely to be retained as well (although the individual TFBS within the CRM may be altered). By applying phylogenetic footprinting to the analysis of closely related species (i.e. 50–100 million years of separation for vertebrates), it becomes possible to concentrate predictions within a subset of regions in the conserved segments of genes. Improved specificity is balanced against the reduced sensitivity imposed by any filter.

Once predictions of regulatory sequences have been made, laboratory validation is required to confirm regulatory function. One of the most widely used methods for validating computational predictions of regulatory regions are reporter gene assays in a cell culture model system [25]. A fusion construct of the predicted regulatory sequence and a reporter gene with a basal promoter in a plasmid is transiently transfected into cells, and the reporter gene activity is measured to determine the regulatory impact that the tested sequence exerts. It is feasible to conduct larger-scale experiments to investigate functional properties of panels of candidate CRMs and promoters within cells. Cooper *et al* performed a large screen of promoter activity in 16 cell lines on all predicted promoters in the 1% of the human genome targeted for in depth annotation by the ENCODE Project [26]. Similarly, relatively large-scale *in vivo* enhancer studies have been performed using highly conserved (human to fish) sequences driving reporter gene expression in transgenic mouse embryos, leading to the identification of 75 forebrain-specific enhancers [27]. Kappen *et al.* analyzed the regulatory controls for lsl, a LIM/homeodomain transcription factor, by inserting randomly sheared 8–10 kb fragments from the lsl genomic locus into reporter constructs and testing for expression both *in vitro* and *in vivo* [28]. Using a single copy insertion mouse transgenesis procedure, the Pleiades Promoter Project evaluated over 100 candidate regulatory sequences for the capacity to direct selective patterns of reporter gene expression in the developed brain [29]. The development of higher-throughput approaches to verify enhancer and promoter function has been a focus of recent efforts to annotate vertebrate genomes.

The properties of skeletal muscle CRMs have been widely studied, but relatively few novel functional CRMs have been described since CRM prediction methods have emerged. To quantify the performance of CRM prediction methods requires a new body of reference data. We generated predictions of CRMs with three published methods and assessed the predictive benefit of sequence conservation and annotation of the expression patterns of proximal genes. We employed LRA, MSCAN, and ClusterBuster to scan the human genome for putative skeletal muscle regulatory regions, and tested a subset for the capacity to drive reporter gene expression in a selective manner in the C2C12 cell skeletal muscle differentiation assay. We compare the reporter gene expression in immature myoblasts against expression in mature myotubes, as well as in a fibroblast cell line. Based on the outcomes of the analysis, we define additional properties of sequence composition that are predictive of function and establish a new reference collection for the continuing development of predictive methods.

## Methods

### Human Genome Search Regions

Promoter regions are identified following the procedure described for the oPOSSUM database [30]. The oPOSSUM database contains the set of genes identified as being in one-to-one human and mouse ortholog pairs based on annotations in EnsEMBL v. 41 and UCSC hg18/mm8 whole genome alignments. For each ortholog pair, 10 kb upstream and downstream of a TSS is searched for CRMs. All noncoding regions are included in the search, including intergenic regions, introns, and untranslated regions (UTR) of exons; protein coding portions of exons are excluded. Any noncoding region that constitutes a portion of a coding exon in an alternative transcript is removed from the selection process. All alternative transcription start sites (TSS) supported by either human or mouse Fantoms3 CAGE evidence were identified and 50 bp on either side of each TSS was excluded [31].

### Muscle *cis*-Regulatory Module Prediction

CRM prediction tools were used to search for muscle-specific regulatory modules within the specified genome sequences. Logistic Regression Analysis (LRA), MSCAN, and ClusterBuster were applied to the human genomic sequence regions specified above [15], [18], [19]. The input TFBS motif models were taken from JASPAR, a database of transcription factor binding site profiles [32]. The models used were MEF2A (MA0052), SRF (MA0083), MYF (MA0055), TEAD (MA0090), and SP1 (MA0079); TFs with described key roles in muscle-specific gene expression. Predicted CRMs composed entirely of SP1 TFBS were excluded.

### Conservation Analyses

The candidate regions were analyzed for conservation based on phastCons scores (generated with 28 placental mammal genomes) obtained from the UCSC Genome Annotation system [33]. For a region to be classified as conserved, the presence of at least one sub-region with phastCons scores of 0.7 or greater over 20 bp is required. For each region, both the mean and the maximum phastCons scores were calculated and sub-regions with phastCons scores over 0.7 were extracted and the ratio of the length of these sub-regions over the total length of the region calculated. For phylogenetic depth evaluation, three sets of human phyloP scores (generated with 46 vertebrates, 46 placental mammals and 46 primates; database version hg19) were obtained from the UCSC Genome Annotation system.

### MyoD ChIP-Seq Data

The ChIP-Seq peak locations for MyoD binding regions in the mouse genome were obtained from http://www.cs.washington.edu/homes/ruzzo/papers/DevCell/2010a/, the companion web resource to the reference publication [34].

### Histone Modification ChIP-Seq Data

C2C12 cell ChIP-Seq peak locations for H3K4me1/2/3, H3K9me3, H3K9Ac, H3K18Ac, H3K27me3, and H4K12Ac annotated by Asp *et al.* were downloaded from the NCBI GEO database (GSE25308; [35]).

### TFBS Profile Similarity Comparison

*MatrixAligner* was used to calculate the profile similarity of two TFBSs [36]. This program generates scores from 0 to 2, where a score of 2 indicates complete identity between two matrices being compared.

### Cell Culture

Mouse C2C12 myoblasts (ATCC CRL-1772; American Type Culture Collection; Manassas, VA, USA) and mouse NIH-3T3 fibroblasts (ATCC CRL-1658; American Type Culture Collection; Manassas, VA, USA) were maintained in Dulbecco's modified Eagle's medium, supplemented with 10% (v/v) heat inactivated fetal bovine serum, 100 U/ml penicillin, and 100 µg/ml streptomycin. The cultures were grown at 37°C and 5% CO_2_. Differentiation of myoblasts into myotubes was induced by transferring C2C12 cells to differentiating media consisting of 2% (v/v) horse serum, 100 U/ml penicillin, and 100 µg/ml streptomycin. The media and reagents for cell culture were obtained from Gibco-Invitrogen (GIBCO-Invitrogen Canada, Canadian Life Technologies, Burlington, ON, Canada).

### Plasmids and Cloning

Primer3 was used to design the flanking primers for each predicted CRM for PCR [37]. After performing PCR with the designed primers (synthesized by Invitrogen Coporation (Carlsbad, CA, USA)), 20 ng of each PCR product was pooled, which were then purified using the PCR purification kit (NEB, Mississauga, ON, Canada) and subcloned into the pGL-3 promoter luciferase vector (Promega; Fisher Scientific, Nepean, ON, Canada) via Kpn I and Bgl II restriction enzymes sites. Restriction digest was performed overnight at 37°C. Post-digestion, the vector was dephosphorylated with calf intestinal alkaline phosphatase (NEB, Mississauga, ON, Canada). The restriction enzyme-digested PCR products and the vector were gel-purified using QIAquick gel extraction kit (Qiagen Inc. Mississauga, ON, Canada) and ligated using T4 DNA ligase (NEB, Mississauga, ON, Canada).

A set of control clones and a sample of the library were prepared. Constructs were transformed into sub-cloning efficient DH5α chemically competent *E .coli* cells (GIBCO Invitrogen Canada, Canadian Life Technologies, Burlington, ON, Canada) via heatshock at 42°C and plated on LB agar plates containing 100 µg/ml of Ampicillin for preliminary bacterial colony screening. Colonies were picked and inoculated overnight in 3 ml LB broth with ampicillin. Plasmids were prepared using QIAprep Spin Miniprep Kit (Qiagen Inc. Mississauga, ON, Canada). Sequence confirmation was performed by the CMMT/CFRI DNA Sequencing Core Facility.

### High-throughput Screening of Clone Libraries

Large-scale transformation, colony picking, miniprep, and sequencing reactions with the constructs were performed (Genome Science Centre, Vancouver, BC, Canada). 1 µl of ligation mix was transformed by electroporation into *E. coli* DH10B T1 resistant cells (Invitrogen). Transformed cells were recovered using 1 ml of SOC medium and plated onto 22 cm×22 cm agar plates (Genetix) containing 100 ug/ul ampicillin. Bacterial colonies were picked from the agar plates and arrayed into 384-well microtiter plates (Genetix) using a QPIX automated colony 15 picker (Genetix). Plasmid preparations were performed via an alkaline lysis protocol. DNA sequencing reactions were prepared using a Biomek FX workstation (Beckman-Coulter) and performed using BigDye 3.1 (Applied Biosystems). Analysis of the resulting sequences to the target DNA regions was performed with AlignX from the Vector NTI software (Invitrogen).

### DNA Concentration Measurement and Normalization

Concentration of the plasmid products was quantified using Picogreen assays (GIBCO-Invitrogen Canada, Canadian Life Technologies, Burlington, ON, Canada) via fluorescence measurement with a POLARstar Omega microplate reader (BMG Labtech; Fisher Scientific, Nepean, ON, Canada). All DNA samples were normalized to 100 ng/µl per well.

### Transfection and Reporter Gene Assays

Two sets of C2C12 myoblasts and one set of NIH-3T3 fibroblasts were seeded in 96-well plates at a density of 6000 cells per well. The myoblasts were divided into two sets so that one set could be harvested as myoblasts, while the other set could be differentiated into myotubes prior to harvest. After 24 h (at 70% confluency) in growth media, the cells were transfected with 200 ng of a pGL3-promoter firefly luciferase plasmid construct and 20 ng renilla phRL-TK internal control luciferase plasmid (Promega, Madison, WI) using Lipofectamine 2000 according to the manufacturer's protocol (GIBCO-Invitrogen Canada, Canadian Life Technologies, Burlington, ON, Canada). At 24 h post-transfection, the myoblast C2C12 set and the NIH-3T3 fibroblasts were harvested and luciferase activity measured using the Dual-Luciferase Reporter Assay System (Promega, Madison, WI) and a POLARstar Omega microplate reader (BMG Labtech; Fisher Scientific, Nepean, ON, Canada). The final set of C2C12 myoblasts was switched to differentiating media 24 h after transfection, and incubated for 96 h for differentiation into myotubes. For each clone, duplicate transfections (technical replicates) were performed. The reporter gene activity assays were carried out in two phases. In phase 1, all plasmid constructs were tested in the three cell types. In the second phase, only myotube and myoblast activities were assessed.

### Data Analysis

The following terminology will be used when discussing the experimental data:

- Clone: a single clone bacterial colony with a homogeneous insert sequence
- Plasmid prep: plasmid extraction from a single bacterial culture
- Insert sequence: the genomic region introduced into a plasmid
- Insert plasmid: the vector plasmid containing a sequence of interest
- Clone replicates: replicated experiments using the plasmids from the same clone but from different plasmid preps (i.e. independent DNA preparation)
- Insert replicates: experiments using plasmids recovered from different bacterial clones but sharing the same insert sequence
- Technical replicates: replicated experiments using plasmids from the same DNA preparation

All statistical analyses were done using the R software [38]. The ratios of firefly luciferase expression values over the renilla luciferase expression values were calculated to measure the relative increase of the firefly luciferase activity over the renilla luciferase activity (the internal control for transfection efficiency). Clones that did not produce both firefly and renilla luciferase expression values above the minimum threshold of 1000 luminescence relative light units (LRUs) were marked as failed transfections and removed from subsequent analyses. This heuristic filter was applied to exclude spurious expression ratio measurements, as the ratio of two small values can result in a disproportionately high value, and the VSN procedure intended to mitigate this effect was not sufficient [39]. For those clones where only the firefly luciferase values were above this threshold, the renilla luciferase value was set to the threshold level. This step was designed to minimize the occurrence of large ratios even when the firefly luciferase expression values are near the threshold. The threshold of 1000 LRUs is higher than the median machine background level, which was found to be below 250 LRUs. While this conservative heuristic filter may result in a decrease in sensitivity, the trade-off was deemed acceptable in order to avoid situations where spurious measurements are accepted as false positive results. The expression ratios from the two technical replicates for each clone were averaged, excepting the cases where a replicate transfection failed the minimum expression threshold filter (in such cases the single replicate value was used). The expression ratios obtained for each cell type were normalized using the VSN package. Each clone was treated as an independent sample even though there were in some cases insert replicates. The stochastic variation in the number of insert replicates would otherwise have complicated the analysis. Differential expression between 1) fibroblasts and myotubes, 2) myoblasts and myotubes, and 3) fibroblasts and myotubes groups were determined using the SAM package [40], applying a false discovery rate maximum of 0.05. The two sets of clones selected from phase 1 and phase 2 at the FDR of 0.05 were combined and grouped according to the insert sequence. For each sequence, the number of clones that were identified as showing differential expression was counted, and those sequences with only one supporting clone and/or less than 50% of the available clones identified as positive were excluded from the final set.

## Results

The experimental procedures and analyses presented in this paper consist of four main components: i) computational prediction of muscle-specific CRMs within the human genome; ii) validation of predictions using reporter gene assays and cell culture; iii) assessing performance of CRM prediction tools on the experimentally tested regions; and iv) analysis of the properties of newly validated muscle-specific CRMs relative to the properties of non-active sequences.

### Region Selection

The overall region selection process is illustrated in Supplemental Figure S1 in Text S2. Three sets of genomic sequences were identified for the study of skeletal muscle CRM predictions: (i) background regions randomly selected from conserved regions for control (*background* set); (ii) predicted skeletal muscle CRM regions proximal to skeletal muscle-expressed genes (*muscle* set); and (iii) predicted skeletal muscle CRM regions proximal to genes with no observed link to skeletal muscle (*non-muscle* set). Prediction of CRMs was performed for the *muscle* and *non-muscle* sets, while the *background* sequences were randomly selected from conserved intergenic regions which may or may not contain predicted CRMs. The sets are further described below.

#### Background

A set of 200 regions was selected randomly from intergenic regions within the oPOSSUM conserved sequences (Methods) with high regulatory potential scores [41]. The scores are intended to reflect consistency with the pattern of sequence identity in genome sequence alignments observed in known CRMs, and minimally reflect regions of greater sequence conservation. It is important to note that regions selected from conserved regions of the genome are likely to have distinct properties from regions randomly selected from the whole genome.

#### Muscle

Gene expression profiles associated with elevated expression concurrent with C2C12 myoblast-to-myotube differentiation were identified from the literature. Distinct sources of annotated skeletal muscle genes follow. Moran *et al.* performed gene expression profiling using Affymetrix oligonucleotide arrays, and identified 108 genes up-regulated in differentiated myotubes using one-way nested analysis of variance [42]. Tomczak *et al.* profiled expression using Affymetrix GeneChips, from which they identified 447 genes up-regulated in myotubes through hierarchical cluster analysis with CAGED 1.1 software [43]. In a complementary study, Blais *et al.* performed ChIP-chip analysis that identified 198 regions bound by MyoD, myogenin or Mef2 [44]. Kislinger *et al.* examined global proteome changes by tracking the abundance of 1865 proteins through gel-free tandem mass spectrometry in both myoblasts and myotubes, of which 80 were identified as up-regulated in myotubes [45]. The superset of the skeletal muscle genes arising from these studies was compiled. We previously annotated a list of 28 CRMs in 24 human genes for which at least one regulatory region responsible for skeletal muscle expression had been confirmed experimentally; we refer to this collection as the muscle reference set (a portion of this set was described in [15]; listed in Dataset S1). Combining the superset with the muscle reference set yields 610 unique skeletal muscle-selective genes based on C2C12 experimental data.

Three CRM prediction programs were applied to the 610 sequences and 2,167 putative CRMs were recorded. A total of 518 candidate regions were predicted by more than one program, and high-quality primers for the same experimental PCR settings could be designed for 271 of them using Primer3. Further 220 primers could be designed for 400 randomly selected putative CRMs that were predicted by one program only. In the end, 384 candidate regions were selected for PCR amplification. For the muscle reference set, albeit highly circular due to the use of most of the sequences in parameter training for the published methods, we assessed the number of known CRMs detected by each program: Cluster-Buster detected 16, LRA detected 13, and MSCAN detected 10.

#### Non-muscle

The set of genes represented in the oPOSSUM database excluding the 610 muscle genes were similarly scanned with the three prediction tools. These candidate CRMs were screened to remove any overlap with CRMs included in the *muscle* set or the *background* set.

### Validation of CRM Activity in Cell Culture

#### Library construction and properties

The above sets of predicted CRMs and background regions were inserted into luciferase reporter gene plasmids and prepared as clone libraries. As the clone recovery process was stochastic, only a subset of the regions was recovered from each library and the number of insert replicates for each candidate CRM was variable. In the end, 355 unique insert sequences were present in 672 tested plasmids, of which 339/355 were successfully aligned to the intended PCR regions. The specific number of recovered candidate regions from each collection (88 *background*, 196 *muscle* or 55 *non-muscle*) is given in Supplemental Table S2 in Text S1; their locations are listed in Dataset S1. A brief discussion on the technical challenges faced is given in Text S3.

#### Validation of the assay

Before proceeding with the experimental validation of the 339 sequences, we first assessed the performance of the dual luciferase reporter assay with known CRMs from muscle-expressed genes (desmin, TN-I) [46]–[48] and non-muscle expressed genes (PAH) [49]. The activity of the pGL3-promoter plasmid served as a negative control, while the PAH sequence was anticipated to function equivalently in differentiated and undifferentiated C2C12 cells. Two independent plasmid preparations were assessed with transfections performed in triplicate. The expression of the reporter gene driven by the muscle CRMs was elevated 5-fold (DES) and 15-fold (TNI) in myotubes relative to myoblasts, while the non-muscle CRM (PAH) was unchanged (Supplemental Figure S2 in Text S2).

#### Reporter expression analysis

**Figure 1:**
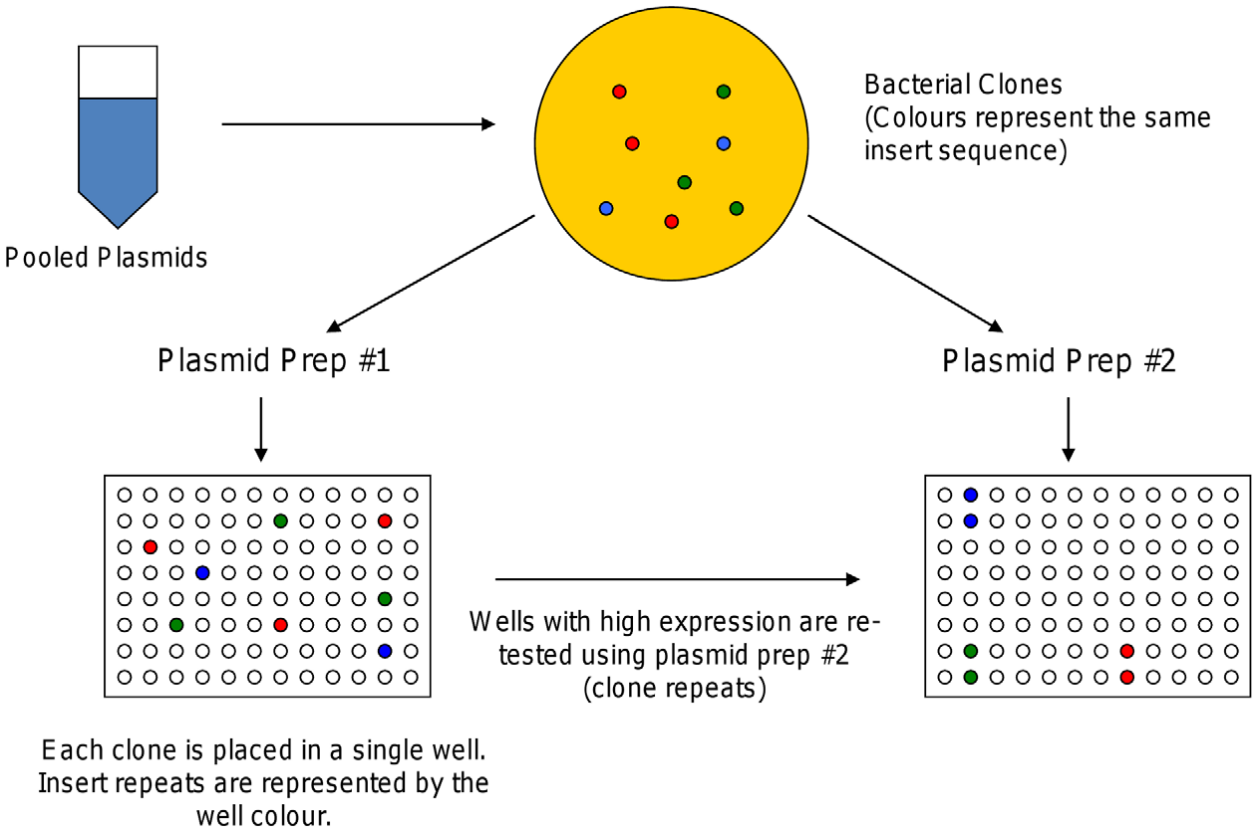
Selection of clones for differential expression analysis. The selection is divided into 2 phases, where the clones selected for Phase 2 are a subset of all clones tested in Phase 1. Phase 1 and Phase 2 samples are from different plasmid preparations.

An overview of the clone production process is presented in Figure 1. The subset of plasmids that selectively directed myotube expression (2-fold increase or elevated based on SAM analysis) in phase 1 was advanced for further analysis in phase 2. In addition, single rows from each of the seven plates used in phase 1 were advanced, in order to assess the reproducibility of results. This selection process resulted in 204 plasmids being advanced. Independent plasmid preparations were used in the second round.

While individual predicted CRM inserts exhibit higher expression than the background controls, the mean expression ratios of the two sets are not significantly different based on a t-test (Supplemental Figure S3 in Text S2). Reporter gene expression increases from myoblasts to myotubes are similar between the two groups, although the predicted CRM inserts exhibit higher variability. Both firefly and renilla raw reporter expression values were lower for the background controls. This characteristic was observed for both phase 1 and phase 2 (independent plasmid preparations).

Using the analysis criteria described in Methods, a set of 19 novel insert sequences was identified as driving selective myotube expression (relative to myoblasts and fibroblasts) (Table 1). These 19 CRMs are hereafter referred to as the validated positive regions. Of the 19 CRMs, 11 were derived from the muscle gene insert set, 4 from the non-muscle set and 4 from the conserved regions control group. Application of the CRM prediction tools to the 4 functional sequences from the control group resulted in 1 putative CRM being identified by ClusterBuster.

**Table 1:** List of genomic regions validated as driving muscle-specific expression.

	Coordinates					Prediction Method		
Set	Chr	Prediction	Insert	Gene Name	Positive Wells	C. Buster	LRA	MSCAN
BG	11	116201218–116201584	116201218–116201584	APOA4; APOC3	4/5			
BG	11	1721407–1721765	1721407–1721765	HCCA2	3/6	C		
BG	11	66008803–66009164	66008803–66009164	DPP3	2/2			
BG	11	71615350–71615709	71615350–71615709	INPPL1	2/2			
NM	15	83185168–83185457	83184998–83185491	ALPK3	2/2	C	L	M
NM	18	40637176–40637555	40637097–40637579	SETBP1	3/4	C	L	M
NM	22	22516591–22516990	22516569–22517062	DERL3; SLC2A11	10/11	C	L	M
NM	22	22883635–22883974	22883584–22884017	CABIN1	2/4	C	L	M
M	1	119250477–119250882	119250477–119250882	TBX15	2/2	C	L	M
M	1	199612030–199612429	199611961–199612457	TNNT2	2/4	C		M
M	2	144878638–144878867	144878533–144878970	ZEB2	2/2	C		M
M	2	88147937–88148156	88147915–88148315	SMYD1	4/4		L	
M	4	37728510–37728799	37728494–37728957	TBC1D1	2/4		L	M
M	6	42106432–42106831	42106373–42106867	CCND3	2/2	C	L	M
M	6	7127465–7127684	7127364–7127817	RREB1	2/4		L	M
M	9	35677988–35678317	35677887–35678364	TPM2	6/9	C	L	
M	14	104259446–104259775	104259362–104259861	INF2; ADSSL1	2/3	C	L	
M	19	3326530–3326884	3326530–3326884	NFIC	4/4		L	M
M	19	54184895–54185244	54184834–54185225	GYS1	2/3	C	L	M

Gene names were chosen for their proximity to the regions of interest (UCSC hg18). ‘Positive Wells’ column shows the number of replicates that were classified as positive out of all replicates for the sequence. Columns ‘C.Buster,’ ‘LRA,’ and ‘MSCAN’ indicate programs which predicted a CRM in the given region.

### Properties of the Validated Positive Set

To identify defining characteristics of the positive regions compared to the non-responding regions, the validated set was subjected to analyses based on sequence and conservation properties.

#### Overrepresented TFBS

The oPOSSUM analysis method was applied to identify TF binding motifs over-represented in the 19 functional CRMs relative to the inactive inserts (Table 2). The top-scoring TFBS in the myotube-directing background regions are those of RREB1, dorsal and NHLH1 [50]–[52]. We could not find any direct link between muscle development and RREB1 in the literature. It is possible that the enrichment is just an artefact due to the RREB1 motif having high information content, which results in infrequent binding site predictions compared to most other motifs. If the foreground regions contain even just a few sites, it can result in high over-representation scores compared to the background set. Dorsal, a Rel TF, is involved in early stages of fly development; however, we could not find any direct role that Rel TFs play in vertebrate muscle development. As there could be other contributing motifs beyond the 5 muscle-linked motifs used the initial CRM prediction methods, further oPOSSUM analyses were carried out: 1) comparing the validated regions from the non-background sets as the test set against the remainder of the non-responding regions of the non-background sets as the control set, and 2) comparing the entire set of validated regions against all inactive inserts. Because all non-background regions are CRM predictions made with the 5 muscle motifs, these motifs were expected to be prevalent in both the positive and non-responding regions. Unexpected motifs present in the positive regions but absent in the non-responding regions could contribute to increased expression in myotubes. If this were the case, these additional motifs would be expected to be overrepresented in the above oPOSSUM analyses. Comparison of the non-background validated regions against the non-responding regions returned MEF2A and NHLH1 as being the most over-represented TFs (Table 3). MEF2A is one of the five muscle TFs used to make the CRM predictions. As not all predictions necessarily contain MEF2A hits, this result indicates the importance of this binding site being present for functional muscle-specific CRM relative to the other four muscle motifs. It suggests that MEF2 binding sites are potential master sites for a subset of active CRMs. NHLH1 is a bHLH TF, the same TF class as the myogenin family, sharing a similar binding profile with this group (normalized score of 1.73 using the *MatrixAligner* program, as explained in the Methods section). The over-representation of this motif may be an indirect marker for Myf, one of the five profiles contributing to the CRM predictions. Comparison of all validated regions against all non-responding regions again returns NHLH1, RREB1 and MEF2A as the most over-represented motifs (Table 4; Supplemental Figure S4 in Text S2).

**Table 2:** Over-represented TFBS in the background regions of the validated set vs. the non-responding background regions (ranked by Fisher p-values).

TF	TF Class	Ctrl gene hits	Ctrl gene non-hits	Target gene hits	Target gene non-hits	Z-score	Fisher score
RREB1	ZnF-C2H2	8	43	3	1	13.29	2.23E-02
Dl	REL	26	25	4	0	5.27	8.04E-02
NHLH1	bHLH	16	35	3	1	7.03	1.14E-01

**Table 3:** Over-represented TFBSs in the non-background regions of the validated set vs. the non-responding non-background regions (ranked by Fisher p-values).

TF	TF Class	Ctrl gene hits	Ctrl gene non-hits	Target gene hits	Target gene non-hits	Z-score	Fisher score
MEF2A	MADS	116	92	13	2	4.48	1.55E-02
NHLH1	bHLH	120	88	12	3	7.98	7.36E-02
Fos	bZIP	181	27	15	0	4.10	1.35E-01

**Table 4:** Over-represented TFBSs in all regions of the validated set vs. the non-responding regions (ranked by Fisher p-values).

TF	TF Class	Ctrl gene hits	Ctrl gene non-hits	Target gene hits	Target gene non-hits	Z-score	Fisher score
NHLH1	bHLH	136	123	15	4	9.79	2.07E-02
RREB1	ZnF-C2H2	50	209	8	11	11.49	2.49E-02
MEF2A	MADS	125	134	13	6	3.38	7.16E-02

#### Sequence composition

The next property examined was the sequence composition of the validated regions. Specifically, we analyzed both the single and dinucleotide composition characteristics of these regions to see if any significant biases could be found compared to the non-responding regions. The Wilcoxon rank sum test was performed to determine if any of these region sets showed significantly different composition characteristics. As shown in Table 5, both the muscle validated regions have higher G/C mononucleotide frequency compared to the non-responding regions; the significance of these differences is supported by the rank sum tests for which most p-values were below 0.05.

**Table 5:** Sequence composition characteristics of the responding regions vs. non-responding regions.

	Responders	Non-Responders	p-value
Muscle Validated	0.54	0.51	4.35E-02
Muscle Reference	0.58	0.51	2.87E-06
Pleiades Curated All	0.55	0.51	1.67E-02
Pleiades Curated Human	0.56	0.51	3.40E-03

GC content; P-values were calculated using Wilcoxon rank sum tests.

We also calculated the G/C and A/T skews in these sequences, but no significant differences in these two measures could be found between the responding regions and the non-responding regions (Supplemental Table S3 in Text S1).

**Figure 2:**
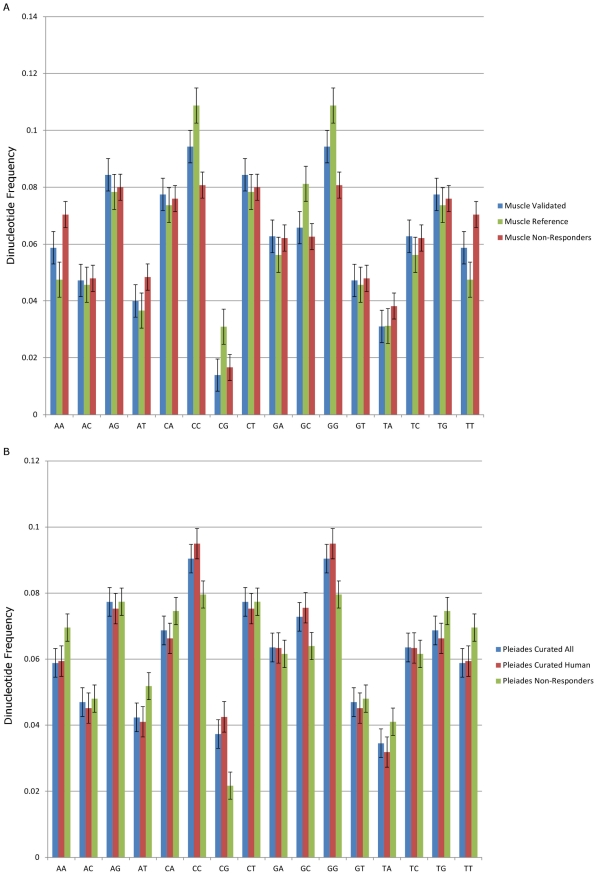
Dinucleotide frequencies in responding regions vs. non-responding regions. a) Muscle Regulatory Regions. Muscle Validated = 19 validated muscle regions in this study. Muscle Reference = 28 muscle reference regions from literature. Muscle Non-Responders = all regions that were tested in this study and found not to drive gene expression. b) Pleiades Curated Regulatory Regions. Pleiades Curated All = 1341 curated regulatory regions from all species. Pleiades Curated Human = 631 curated regulatory regions in humans only. Non-Responders = all regions that were tested and found not to drive gene expression.

To further characterize the sequence compositional differences between the responding and the non-responding regions, we analyzed the dinucleotide compositions of the sequences (Figure 2a). Differences were found in the frequencies of AA, CC and GG, where the responding regions have higher frequencies of CC and GG dinucleotides, and the non-responding regions have higher frequencies of AA dinucleotides. This gives further support to the difference in the G/C compositional characteristics of the responding vs. non-responding regions. The CpG dinucleotide was not enriched in the validated regions and analysis presented below suggests that the enrichment properties are not related to the well-known properties of CpG islands (this point will be further explored below).

To examine whether such biases are present in CRM regions for other tissue types, we performed composition analyses of the curated brain-specific CRM collection from the Pleiades Promoter Project [29]. The goal of this project is to construct human mini-promoters that drive gene expression in specific brain regions. As part of this project, the authors compiled a set of regulatory sequences from 296 genes shown to act as brain-specific CRMs in literature, which they deposited into the PAZAR database [53]. They also performed *in vivo* expression studies of their mini-promoter constructs to identify the regulatory sequences that can drive gene expression specifically in brain regions. Because this data set is comprised of sequences from a number of different species, we performed our analysis on both the entire set and human sequence subset. For the control set to compare against, we use the non-coding human sequences that were tested in the project and found to have no effect on driving gene expression in the mouse brain. Similar to the muscle sequences, the Pleiades brain CRMs display elevated G/C content compared to non-functional sequences (Table 5). As for the dinucleotide frequencies, while not as pronounced in the muscle responders vs. non-responders, for the Pleiades regions, TA, TG and TT dinucleotides occur more frequently in the non-responders (Figure 2b). Lastly, we repeated the analysis for the MyoD ChIP-Seq peak locations identified by Cao *et al.*, and found elevated G/C content in the MyoD-binding regions (Table 6).

**Table 6:** Sequence composition characteristics of the MyoD ChIP-Seq peaks compared against the non-responding regions from this study.

	MyoD ChIP-Seq	Non-Responders	p-value
Myotube	0.54	0.51	1.23E-06
Myoblast	0.54	0.51	6.60E-07

GC content; P-values were calculated using Wilcoxon rank sum tests.

#### Evidence of muscle expression

We attempted to assess if myotube expression data was predictive for functional CRM activity in the results (Supplemental Table S4 in Text S1), however there were insufficient numbers from most datasets to determine significance.

#### Sequence conservation

**Figure 3:**
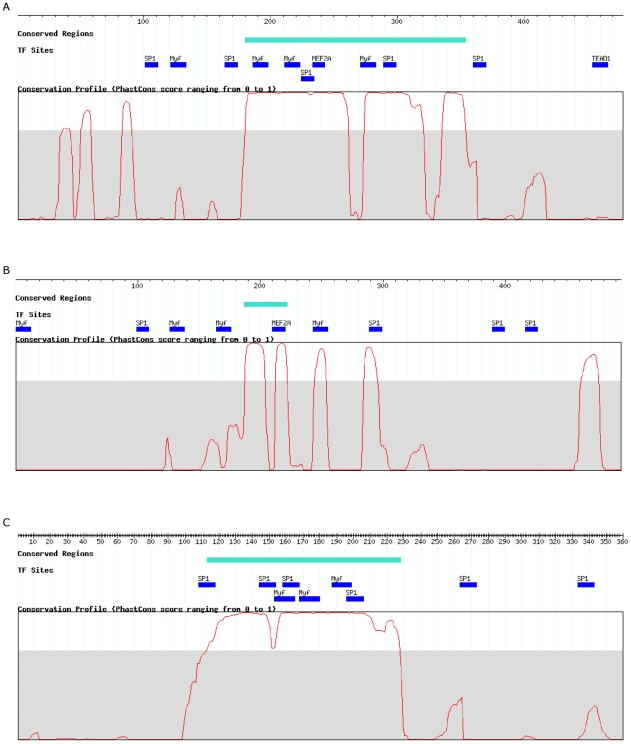
Examples of positive regions. Muscle TFBS hits (threshold of 80%) and the phastCons conservation profile for the region are shown as well. When square brackets are shown, they indicate the original CRM prediction. a) Positive sequence from the muscle set. The muscle-specific TFBS are located in regions of high sequence conservation. b) Positive sequence from the non-muscle. This sequence showed the most consistent increase in reporter expression, with all 12 replicates determined as significantly up-regulated in muscle. c) Positive sequence from the background set. Despite the clear cluster of muscle-specific TFBS located in the region of high sequence conservation, none of the CRM prediction tools could classify this as a muscle CRM.

In selecting candidate skeletal muscle CRMs, we did not incorporate phylogenetic footprinting (sequence conservation). This exclusion allows for a retrospective assessment of the impact of conservation-based filters on the specificity and sensitivity of the predictions. Table 7 gives the comparison of the sequence conservation characteristics of the validated regions versus the non-responding regions. While the average lengths of the PCR amplified inserts averaged ∼400 bp, only a portion of each sequence may be conserved. Global measures may therefore fail to reflect the presence of a locally conserved putative CRM. To alleviate this potential problem, we measured both the mean and the maximum sequence conservation scores for each region and then calculated the mean of these values for each region set. For the validated regions from the non-background sets, we observe both higher mean and maximum phastCons scores compared to the non-responding regions. This observation supports the validity of the widely used approach of applying conservation filters when making predictions for functional genomic regions. While some genome-wide ChIP-Seq studies for TFs have suggested that the conservation of the TF binding regions are limited, it is important to recognize that the TF binding by itself does not necessarily indicate *cis*-regulatory function. Figure 3 presents examples of positive regions from each of the background, non-muscle and muscle sets, representing the different conservation characteristics observed. The performances of the three methods with and without sequence conservation filter are summarized in Supplemental Table S5 in Text S1.

**Table 7:** Sequence conservation based on phastCons scores (28-way Placental Mammals).

Region Set	Mean Score	Avg. Max Score	Conserved Region Make-Up
All Positives	0.20	0.84	18.9%
Positives from Background Set	0.12	0.74	7.9%
Positives from Non-Background Sets	0.22	0.87	21.2%
All Non-Responding Regions	0.17	0.77	15.1%
Non-Responding Regions from Background Set	0.22	0.87	18.4%
Non-Responding Regions from Non-Background Sets	0.16	0.75	14.4%

Column ‘Mean Score’ refers to the overall mean of the mean scores for each region in each set, while ‘Avg. Max Score’ refers to the mean of the highest score for each region in each set. ‘Conserved Region Make-Up’ lists the summed conserved region lengths (identified as sub-regions with phastCons score over 0.7) divided by the sum of the lengths of all regions in each set.

We performed receiver operating characteristic (ROC) analysis for Cluster-Buster, LRA, and MSCAN using the ROCR package in R [54]. The ROC analysis was performed both with and without a conservation filter applied, based on the maximum phastCons scores (Supplemental Figure S5 in Text S2). For true positive regions, we included both the insert sequences that were validated through the reporter expression assays or literature-derived known muscle reference regions exclusive of the skeletal muscle training sets used in the development of LRA and ClusterBuster (see Dataset S1). For negative examples, all predicted and tested CRM regions (*muscle* and *non-muscle sets*) that did not respond to the reporter assays were used. An ROC curve based solely on the conservation filter was also generated. While adding the conservation filter improved the prediction performance for all methods tested, the conservation filter-only results exhibited the best performance, with the AUC of 0.76. However, it is noted that a large percentage of the non-responding regions come from the predictions by the three methods (211 of 295 regions, or 71.5%); as such, this high AUC of 0.76 is not independent of the contributions by the prediction programs. The findings confirm utility for incorporating sequence conservation into the prediction of *cis*-regulatory modules.

#### Promoters and CpG islands

We examined the distances of the regions to the nearest Ensembl-annotated TSSs (Supplemental Table S6 in Text S1). While there was much variability in the distances, with some regions located more than 100 kb away, the responding regions were in general located closer to the TSSs than the non-responding regions (median of ∼1 kb vs. ∼12.5 kb). In order to determine if the responding regions are associated with CpG islands, 1 kb upstream and downstream from each region were searched for UCSC-annotated CpG islands (Supplemental Table S7 in Text S1). While a higher proportion of the responding regions were associated with CpG islands compared to non-responding regions, the difference did not have statistical significance (p-value of 0.13 obtained with Fisher test). Only 2 regions from the validated set were associated with CpG islands (10.5%), while 10 regions from the reference set overlapped with CpG islands in their flanking sequences (35.7%). This increase is likely due to the fact that the reference set regions are more proximal to the TSSs (median distance of 122.5 bp) than the validated set regions (median distance of 4,606 bp), and CpG islands are also typically in the vicinity of TSSs [55]. As evident in Table S7 in Text S1 and Figure 2, the muscle reference regions do display elevated CpG frequency and CpG island proximity, consistent with experimental bias in early promoter analysis for regions proximal to transcription start sites.

#### Phylogenetic depth

Cheng *et al.* performed ChIP-chip analysis of GATA1 binding regions in mouse erythroid cells and observed that most of the GATA1 binding regions contained the canonical binding site motifs [56]. However, they determined that the GATA1 binding motifs in regions associated with high enhancer activity were more evolutionarily conserved compared to those motifs in regions with no identifiable enhancer activities. To evaluate whether this observation holds for the muscle regulatory regions identified in this study, we searched the three region sets for binding site hits with all available vertebrate profiles from the JASPAR CORE collection. When overlapping sites for the same motif were found, only the highest scoring site was kept.

**Figure 4:**
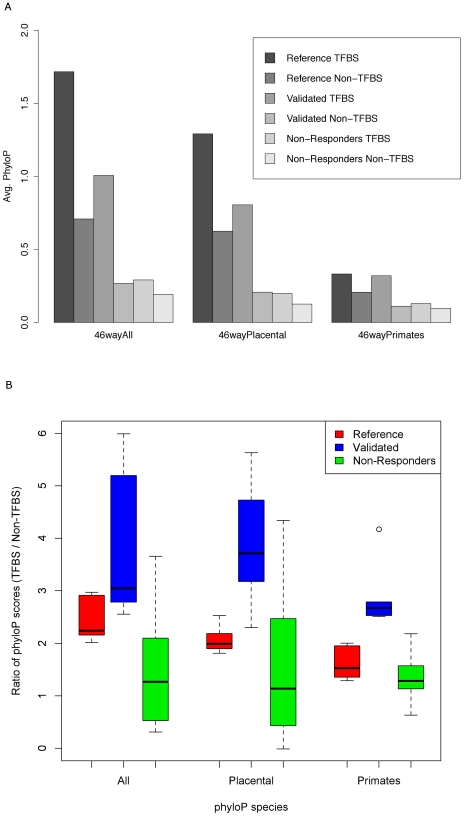
Phylogenetic depth analysis of TFBSs in responding and non-responding regions using phyloP (46-way, hg19). The x-axis grouping indicates the species used to calculate the phyloP scores. TFBSs were searched using all vertebrate profiles from the JASPAR CORE collection using the threshold of 0.8. TFs with at least 2-fold increase in phyloP scores (46wayAll) in the TFBS positions over the non-TFBS positions in the responding regions were identified. The score ratios for these TFs were compared among the three region sets. a) Average phyloP scores for the predicted TFBS positions and non-TFBS positions in each region set. b) Ratios of phyloP scores for the TFBS positions and non-TFBS positions in each region set.

We first calculated the average phyloP scores for the predicted binding site and non-binding site positions in each of the three region sets. The scores for the responding regions are spread over a larger range than the non-responding regions, which also showed the lowest mean score (Supplemental Figure S6 in Text S2). We identified the TFs with predicted binding sites that exhibited at least 2-fold increase in their phyloP scores over the non-binding site positions in each region set, and compared the mean phyloP scores in the predicted sites for these TFs among the three region sets (Supplemental Table S8 in Text S1). The average phyloP scores were significantly higher for the validated and reference sets than for the non-responding set, as confirmed by t-tests. Included in the TFs with 2-fold increase in the validated and reference sets are some of the known muscle-specific TFs, such as MEF2A, Myf, SRF and PBX1 [57]. We combined the list of the TFs with at least 2-fold increase in the validated and the reference set, and compared the ratios of the phyloP scores for the predicted sites for these TFs and the non-binding site positions in each region set (Figure 4). The ratios are significantly higher for the responding regions (confirmed with t-test; p-value of 2.4×10^−4^ for all species; Supplemental Table S8B in Text S1). When phyloP scores are calculated using more closely related species, the p-values become more significant. Non-responding regions do not show such a trend in scores (confirmed with t-test; p-value of 0.76 for all species and primates only).

#### MyoD ChIP-Seq evidence

Cao *et al.* performed a genome-wide ChIP-Seq binding assay for MyoD in C2C12 myoblasts and myotubes [34]. We compared the MyoD peak locations with our three region sets to determine the extent of overlap with each. As we were testing the regulatory effects of human genomic sequences in murine C2C12 cells, we first performed a lift-over of the regions to the mouse genome using the Galaxy service, transitioning from human assembly hg19 to mouse assembly mm9 [58]. Successfully mapped regions were then compared with the MyoD peak locations (Table 8). Whereas only 15.6% of the successfully mapped non-responding regions overlapped with the MyoD peaks in myotubes, 58.1% of the responding regions overlapped with the peaks, which is a significant increase (p-value = 3.0×10^−8^ with Fisher exact test). These observations support the use of ChIP-Seq assay results for achieving improved specificity in the identification of functional regulatory elements.

**Table 8:** Regions overlapping MyoD ChIP-Seq peaks in C2C12 cells.

	Myoblasts	Myotubes	P-value
Responding	27.9% (12/43)	58.1% (25/43)	4.3E-03
Non-responding	11.6% (23/199)	15.6% (31/199)	1.5E-01
P-value	8.4E-03	3.0E-08	

P-values were calculated using Fisher exact tests.

#### Histone modifications

**Figure 5:**
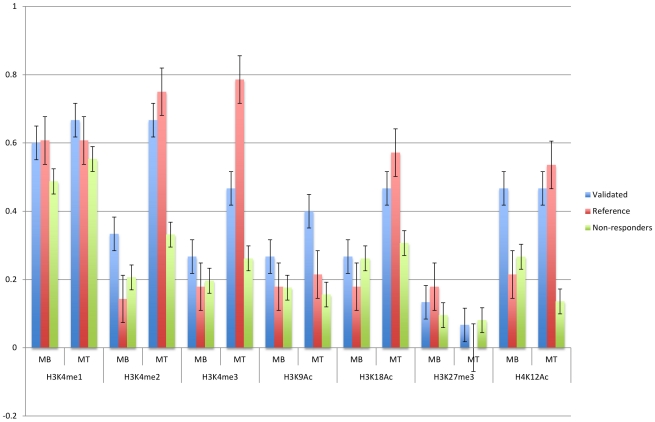
Histone modifications in the responding and non-responding regions. Proportion of the regions that overlap with ChIP-Seq peaks from Asp et al. are displayed. (MB = Myoblasts, MT = Myotubes).

Chromatin conformation changes through histone modifications play an important role in the regulation of gene expression. Acetylation of histone tail residues lead to open chromatin, allowing TFs better access to enhancer regions. Histone methylation has been associated with both activation and repression depending on which residues are modified. In order to examine the epigenetic changes associated with myotube formation, Fischer *et al.* performed a ChIP-chip study of major histone modifications (H4ac, H3ac, H3K4me2/3) in C2C12 cells, from which they observed that H3K4me2 (when combined with other acetylation in the same region) and H4ac were frequently associated with elevated expression [59]. Asp *et al.* performed a more comprehensive ChIP-Seq study in C2C12 cells, where they identified the locations of H3K4me1/2/3, H3K9me3, H3K9Ac, H3K18Ac, H3K27me3, H4K12Ac, and PolII in both myoblasts and myotubes [35]. Using the subsets of the validated, reference and non-responders that were successfully mapped to the mouse genome, we examined the extent of overlap between these regions and the modified histone peaks for H3K4me1/2/3, H3K9Ac, H3K18Ac, H3K27me3 and H4K12Ac (Figure 5). We observed increases in the proportion of the combined set of responding regions that overlapped with H3K4me2 (p-value = 3.5×10^−6^ with Fisher exact test), H3Kme3 (p-value = 2.7×10^−5^), and H3K18ac (p-value = 3.4×10^−3^) peaks in myotubes compared to myoblasts, whereas the non-responding regions did not show as large increases (p-values for H3K4me2: 6.5×10^−3^, H3Kme3: 1.5×10^−1^, and H3K18ac: 3.7×10^−1^). As the responding regions act as enhancers in myotubes, such observations point to these histone marks as being activating. While not statistically significant, increases in H3K12Ac peak overlaps were observed in responding regions (p-value = 7.8×10^−2^), whereas decreases were observed in non-responding regions (p-value = 1.7×10^−3^). Differences between the properties of the validated and reference regions were observed in some cases (e.g. H4K12Ac), which may reflect the previously mentioned promoter proximity of the later set. There is some decrease in the number of responding regions with H3K27me3 marks from myoblasts to myotubes (p-value = 5.8×10^−2^), whereas there is little change in the non-responding regions (p-value = 7.2×10^−1^). H3K27me3 histone marks are known to play important roles in repression of muscle-specific genes in proliferating cells. If we look at the reference set and the validated set separately, we observe that most only the reference regions show a decrease in the H3K27me3 peaks with borderline statistical significance (p-value = 5.1×10^−2^).

## Discussion

We generated genome-wide predictions of muscle-specific CRMs using three CRM prediction programs, including Cluster-Buster, LRA and MSCAN. Based on the predictions, 339 candidate sequences were tested for CRM activity using promoter-reporter gene assays in a cell culture model of skeletal muscle development, of which 278 were successfully transfected into cells and had reporter expression measurements taken. The validation process revealed 19 myotube-restricted promoter-enhancing sequences. In addition to the known enrichment for sequence conservation of functional CRMs, phylogenetic depth analysis revealed that the individual TFBSs display even higher sequence conservation than the surrounding sequence. The active CRMs exhibited elevated G/C mononucleotide content indicating the value for including sequence composition measures in the implementation of future methods. Comparison of the ChIP-Seq results for MyoD and histone modification marks in C2C12 cells with the identified enhancing sequences further supports their recognized utility in the detection of active, functional CRMs.

The performance of the CRM prediction programs used in this paper was not sufficient for genome annotation. The poor performance is likely reflective of the incomplete information presented for the prediction – the primary sequence and sequence conservation data does not convey information about the three dimensional properties of the nucleus nor the epigenetic state of the chromatin [60]–[63]. As evidenced by the significant increase in the proportion of responding regions that overlap with MyoD and histone modification peaks from ChIP-Seq studies, incorporating the results from ChIP-Seq assays for the relevant TFs, co-activating proteins or histone modification marks can improve the specificity of the predictions. In order for such data to be useful, data needs to be generated for each tissue type analyzed, as CRMs are anticipated to be differentially marked when activated. At this time, there is an insufficient amount of such large scale data available to make this a feasible strategy for many tissue types, but more complete data may become available as the costs of experiments come down and sensitivity increases. Ultimately an intersection of computational and experimental methods will be required for the highest quality annotation of CRMs.

A fundamental question arising out of the work reported here is why methods that appeared to be doing well for skeletal muscle CRM discovery failed to demonstrate strong predictive capacity in application here. One key reason may be driven by selection bias for laboratory studies. The reports of CRMs from individual gene studies may in many cases have been influenced by the identification of muscle-related motifs in the available genomic sequences. Due to the selective publication of those sequences showing positive expression, the relative importance of motif enrichment may have been over emphasized. Another key limitation is that most of the methods generate sufficiently high false prediction rates that the reliability of any specific set of predictions is unlikely to be high. The results here demonstrate the driving need for experimental validation of computational predictions whenever feasible.

One striking observation emerging from this study is the enrichment of G/C mononucleotides in the CRMs, observed both in the new muscle set as well as the brain-directing CRMs from the Pleiades Project [29]. The potential contribution of compositional properties to regulatory regions has been previously explored, including a statistical method for CRM prediction [64] and a recent approach from Evans to classify CRM-containing regions into compositional subsets of genome sequences [65]. These approaches and the data presented here are independent of the long-recognized role of CpG islands in demarcating promoter-containing regions and the influence of CpG enrichment on motif over-representation [66], [67]. While there have been prediction methods released, such as Stubb, EMMA and PhylCRM, that directly incorporate phylogenetic footprinting in order to reduce the false positive rate of their predictions [21], [22], [68], the joint incorporation of nucleotide composition properties and sequence conservation remains to be explored.

The outcomes of this paper include both a novel set of 19 skeletal muscle-directing CRMs for use in future machine learning procedures and the specific call for the inclusion of nucleotide composition properties in the next generation of tools.

## Supporting Information

Dataset S1 Contains the detailed list of the genomic regions tested.
(XLSX)

Text S1 Contains all the supplemental tables referenced in the manuscript.
(DOC)

Text S2 Contains all the supplemental figures referenced in the text.
(DOC)

Text S3 Contains the supplemental notes on technical experimental issues.
(DOC)
