# Supplementary material for: Validation of Skeletal Muscle cis-Regulatory Module Predictions Reveals Nucleotide Composition Bias in Functional Enhancers
Source: PLoS Comput Biol. 2011 Dec 1;7(12):e1002256. doi: 10.1371/journal.pcbi.1002256 (PMC3228787; doi:10.1371/journal.pcbi.1002256)
Supplement: Text S1 — Contains all the supplemental tables referenced in the manuscript. (DOC) [file pcbi.1002256.s002.doc]

**Supplemental Tables**

**Table S1. List of some of the published CRM prediction programs.**

| Name | Method | Author |
| --- | --- | --- |
| LRA | Logistic regression analysis | Wasserman and Fickett |
| MSCAN | Motif-specific p-values | Johansson et al. |
| MCAST | Hidden Markov model | Bailey and Noble |
| Cister | Hidden Markov model | Frith et al. |
| COMET | Hidden Markov model | Frith et al. |
| Cluster-Buster | Hidden Markov model | Frith et al. |

**Table S2. Number of candidate regions.** The number of candidate regions that were actually cloned and tested is lower than the number of candidate regions computationally identified and allocated due to the random selection process.

| Region Set | # Samples Allocated | # Cloned and Tested | # Viable Clones | # Validated as Positive | # Validated / # Viable Clones (%) |
| --- | --- | --- | --- | --- | --- |
| Background | 192 | 88 | 55 | 4 | 7.3 % |
| Non-muscle | 96 | 55 | 37 | 4 | 10.8 % |
| Muscle | 384 | 198 | 186 | 11 | 5.9 % |

**Table S3**. GC and AT skews of the responding regions vs. non-responding regions. The skew values were calculated by: GC Skew = (|G| - |C|) / (|G| + |C|), and AT Skew = (|A| - |T|) / (|A| + |T|).

|  | GC Skew | | | AT Skew | | |
| --- | --- | --- | --- | --- | --- | --- |
|  | Responders | Non-Responders | p-value | Responders | Non-Responders | p-value |
| Muscle Validated | 0.122 | 0.099 | 0.13 | 0.091 | 0.101 | 0.64 |
| Muscle Ref | 0.109 |  | 0.18 | 0.116 |  | 0.49 |
| Pleiades Curated All | 0.113 | 0.093 | 0.26 | 0.094 | 0.084 | 0.82 |
| Pleiades Curated Human | 0.123 |  | 0.08 | 0.096 |  | 0.76 |

**Table S4**. The distribution of the regions in the muscle set according to the evidence source for muscle expression. For each muscle gene, there can be multiple candidate regions selected.

| Evidence Source | # Genes | # Included in Candidate Regions | # Regions Included in the Positive Set |
| --- | --- | --- | --- |
| Blais | 46 | 58 | 4 |
| Emili | 80 | 105 | 2 |
| Moran | 108 | 69 | 3 |
| Tomczak | 447 | 596 | 8 |

**Table S5.** Comparison of the five CRM prediction programs. The five programs were tested on the 278 successfully cloned sequences and on the previously collected muscle reference regions. For the column ‘Programs’, CF refers to the application of additional conservation filter with the same parameters as used in Table 4.

| Programs | Validated Regions | | Non-Responding Regions | | Muscle Reference Regions  (28 regions) |
| --- | --- | --- | --- | --- | --- |
|  | Background (4 regions) | Non-background (15 regions) | Background  (51 regions) | Non-background  (208 regions) |  |
| Cluster-Buster | 1 | 11 | 8 | 143 | 16 |
| Cluster-Buster + CF | 0 | 8 | 5 | 72 | 16 |
| LRA | 0 | 13 | 2 | 136 | 13 |
| LRA + CF | 0 | 8 | 2 | 67 | 13 |
| MSCAN | 0 | 11 | 1 | 131 | 10 |
| MSCAN + CF | 0 | 6 | 1 | 64 | 10 |

**Table S6**. Distances to the nearest annotated transcription start sites (Ensembl v61). All units are in base pairs.

| Region | Min. | Median | Mean | Max. |
| --- | --- | --- | --- | --- |
| Muscle Validated | 100 | 4,606 | 14,699 | 122,235 |
| Muscle Reference | 8 | 122.5 | 1,506 | 15,000 |
| Combined Responders | 8 | 928 | 6,839.4 | 122,235 |
| Non-Responders | 1 | 12,536 | 80,198.7 | 810,495 |

**Table S7**. Regions associated with CpG islands. 1 kb upstream and downstream from each region was searched. CpG island annotations are from the UCSC Genome Browser (hg19).

| Region | # with CpG | # without CpG |
| --- | --- | --- |
| Muscle Validated | 2 / 19 (10.5 %) | 17 / 19 (89.5 %) |
| Muscle Reference | 10 / 28 (35.7 %) | 18 / 28 (64.3 %) |
| Combined Responders | 12 / 47 (25.5 %) | 35 / 47 (74.5 %) |
| Non-Responders | 45 / 269 (16.7 %) | 224 / 269 (83.3 %) |

**Table S8**. Comparison of the mean phyloP scores in the three region sets for profiles with at least 2-fold increase in phyloP scores for predicted TFBS positions vs. non-TFBS positions.

1. Mean phyloP scores for TFBS positions in each region set.

| PhyloP species | Validated | Reference | Non-Responders |
| --- | --- | --- | --- |
| All | 1.15 | 1.77 | 0.49 |
| Placental | 0.78 | 1.51 | 0.33 |
| Primates | 0.32 | 0.45 | 0.21 |

1. P-values from t-test results between each region set (alternative hypothesis: true difference in means is not equal to 0)

| PhyloP Species | Validated vs. Non-Responders | Reference vs. Non-Responders | Validated vs. Reference |
| --- | --- | --- | --- |
| All | 2.39E-04 | 8.14E-09 | 2.06E-03 |
| Placental | 2.12E-05 | 7.05E-09 | 1.28E-07 |
| Primates | 2.86E-07 | 9.58E-08 | 8.43E-09 |

1. TFBS profiles with greater than 2-fold increase in average phyloP scores between predicted TFBS and non-TFBS positions

| Region Set | TF Names |
| --- | --- |
| Validated | Sox5, HOXA5, Prrx2, RELA, Myf, FOXF2, NR2F1, MEF2A, NFYA, Arnt::Ahr, ZEB1, Foxa2, Nkx3-2, CREB1, NHLH1, PBX1, TLX1::NFIC, Myc, GABPA, Ar |
| Reference | Myf, TEAD1, MEF2A, ZEB1, SRF, NFIL3, PBX1, Pdx1, ARID3A, Sox5, CREB1, Pax5, FOXI1, IRF1, Sox2 |
| Common (Intersection) | Sox5, Myf, MEF2A, ZEB1, CREB1, PBX1 |
