# Supplementary material for: Validation of Skeletal Muscle cis-Regulatory Module Predictions Reveals Nucleotide Composition Bias in Functional Enhancers
Source: PLoS Comput Biol. 2011 Dec 1;7(12):e1002256. doi: 10.1371/journal.pcbi.1002256 (PMC3228787; doi:10.1371/journal.pcbi.1002256)
Supplement: Text S2 — Contains all the supplemental figures referenced in the text. (DOC) [file pcbi.1002256.s003.doc]

**Supplemental Figures**

**Supplemental Figure S1.** The overview of the region selection pipeline.


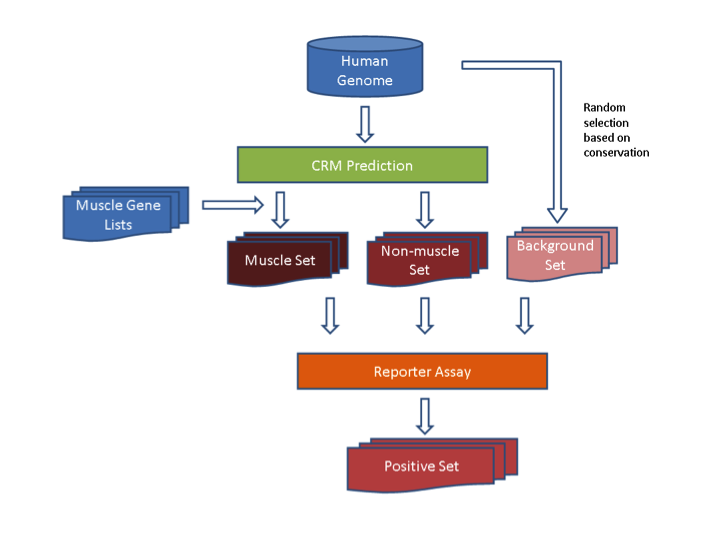


**Supplemental Figure S2.** Reporter expression for known muscle-specific and non-muscle specific enhancer regions in myotubes vs. myoblasts. Only those constructs with known muscle-specific enhancers show marked increase in expression over the empty vectors. Expression ratios are calculated by dividing firefly luciferase reporter expression values by renilla luciferase reporter expression values. The error bars represent the standard error.


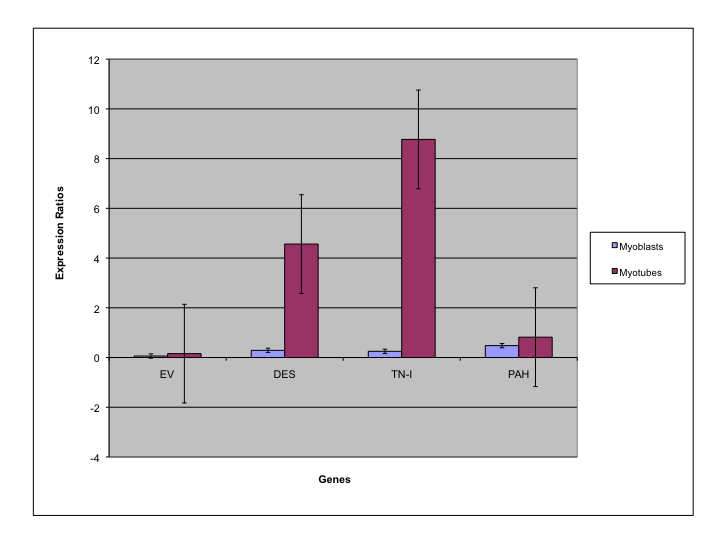


**Supplemental Figure S3.** Comparison of the reporter expression levels between background regions and non-background regions in the validated positive set.

1. Ratios of firefly and renilla luciferase reporter expression levels of the background regions vs. non-background regions in the validated positive set (myotubes). The difference between the two region sets is not significant.


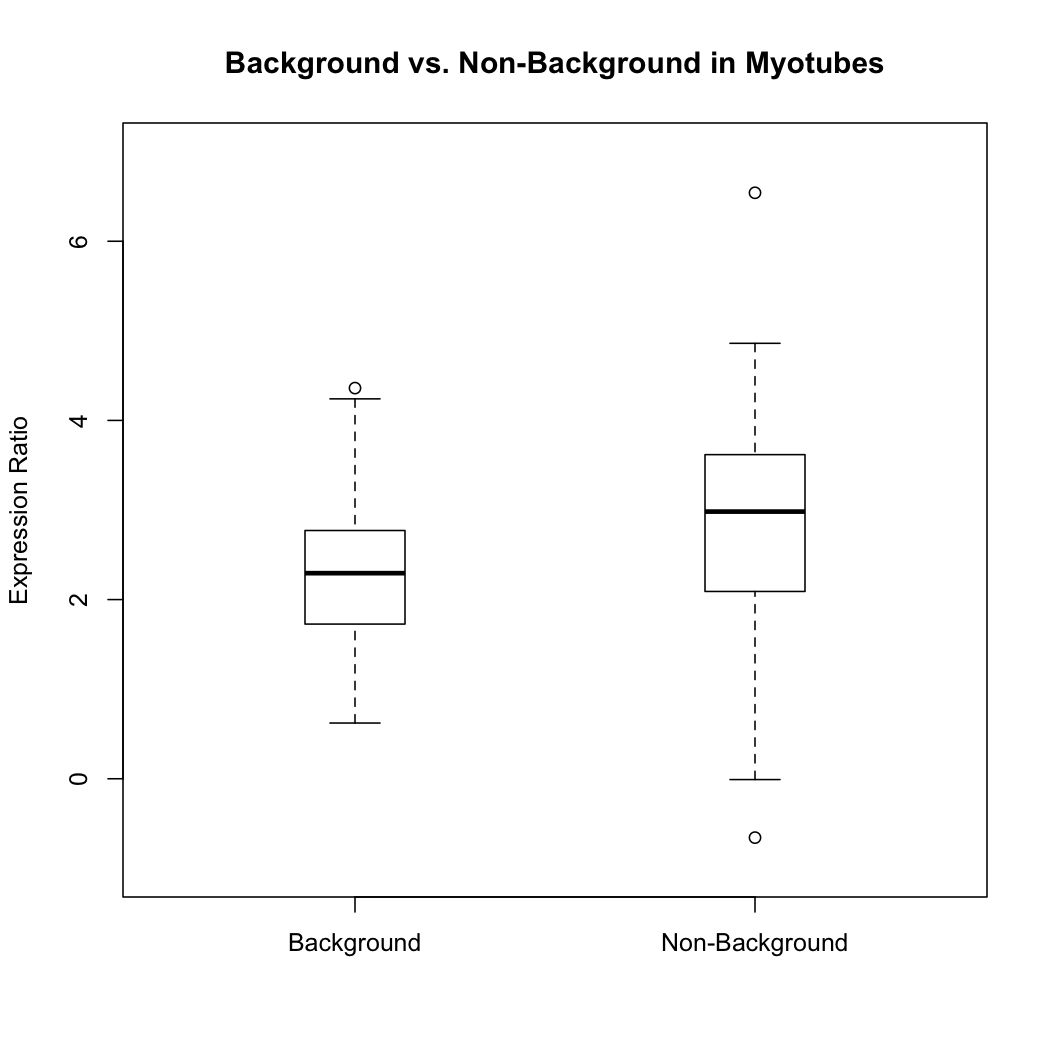


1. Difference in expression ratios between myoblasts and myotubes. While non-background regions show higher spread in expression level differences than the background regions, the average values are not significantly different between the two.


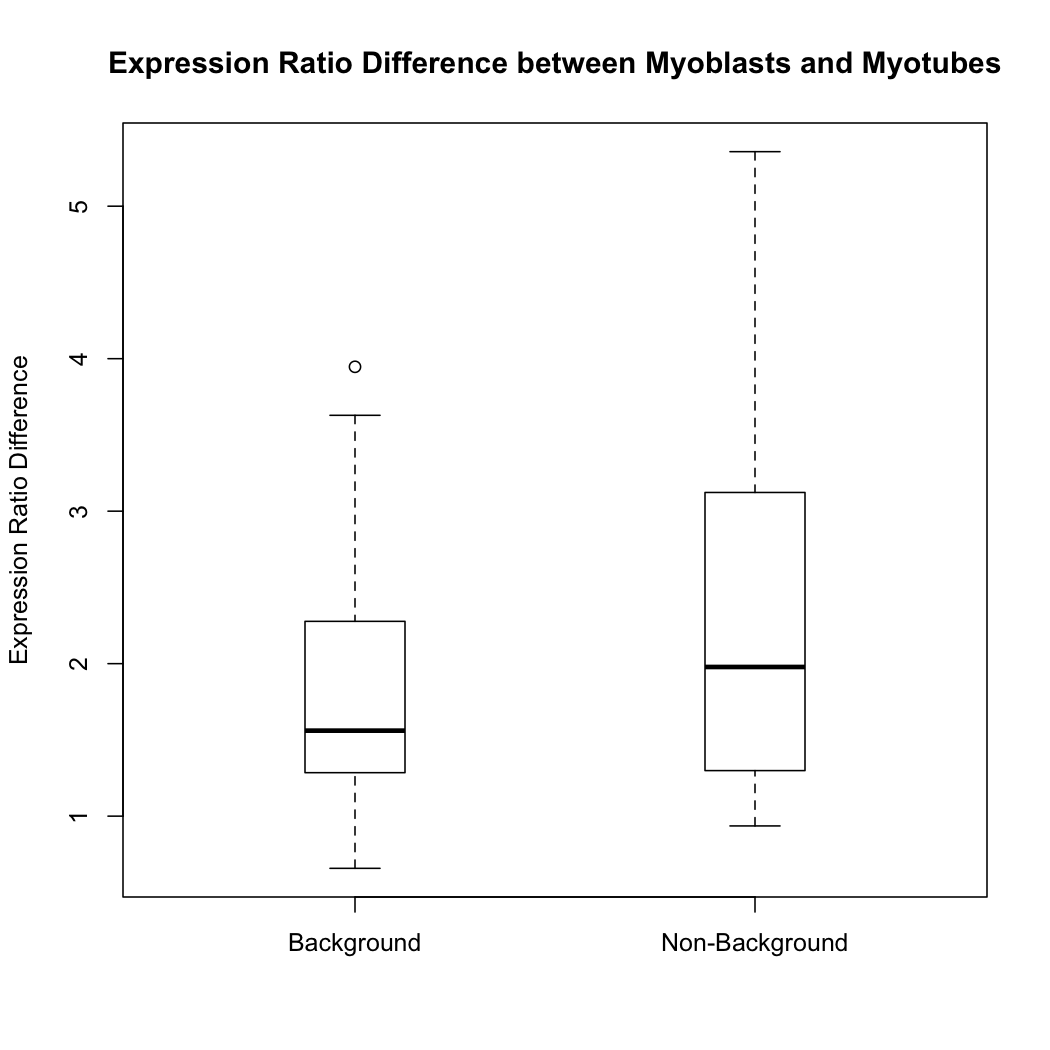


1. Firefly and renilla luciferase reporter expression levels (log2 scale) of the background regions vs. non-background regions in the validated positive set. Non-background regions show significantly higher expression levels than background regions. The difference between the mean firefly activity and renilla activity is also higher for the non-background regions. (BG = Background; Non-BG = Non-Background)

**
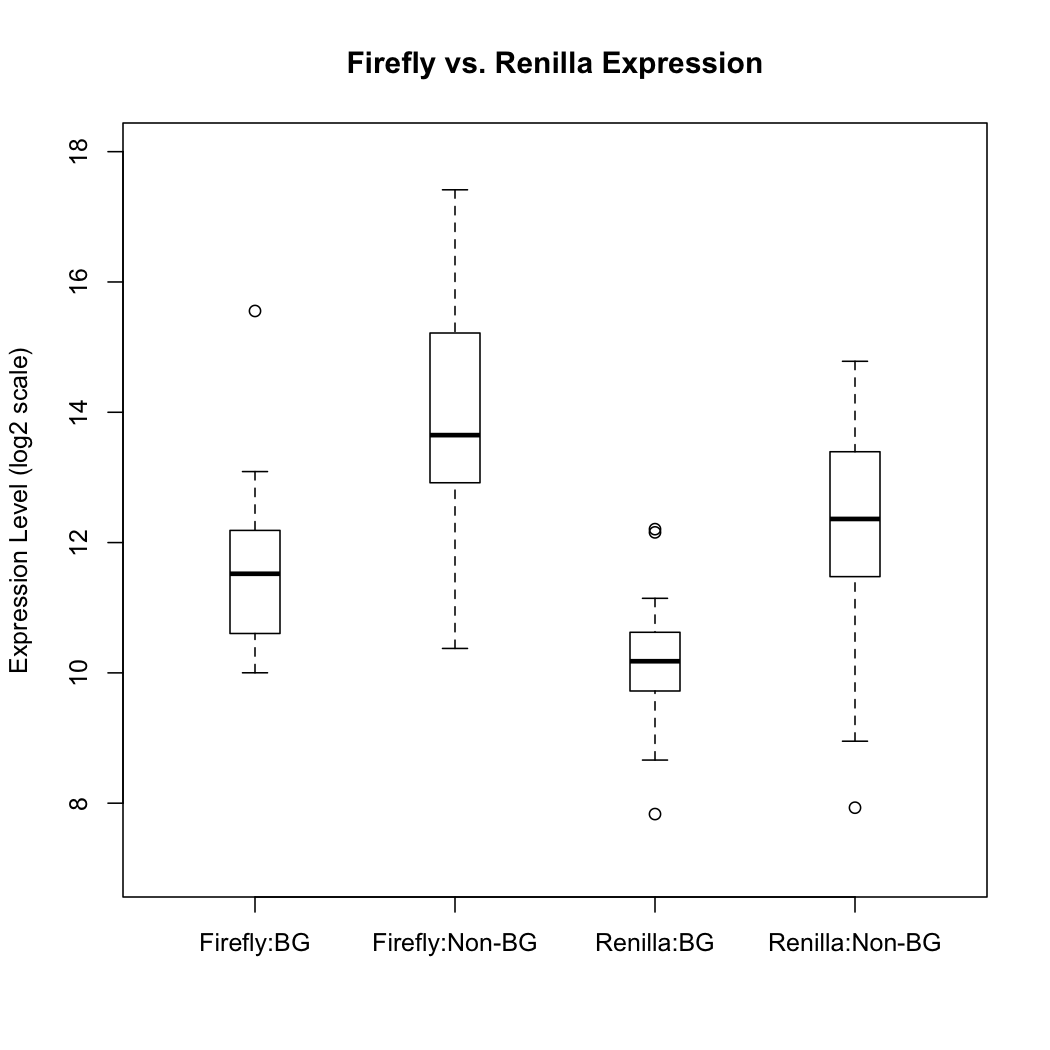
**

**Supplemental Figure S4.** Logos of the 5 muscle TFBSs (SRF, MEF2A, Myf, TEAD, and SP1) used for CRM prediction and 2 additional TFBSs (RREB1, NHLH1) found to be overrepresented in the validated set.


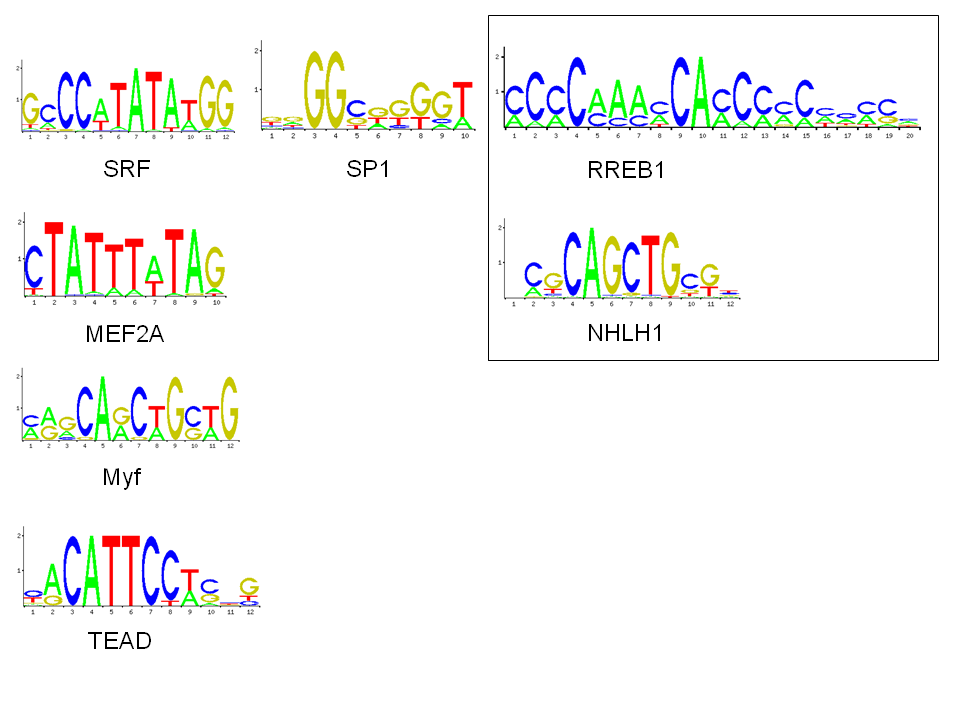


**Supplemental Figure S5.** ROC Analysis of the conservation filter and the 4 CRM Prediction Programs. The conservation filter analysis is based on phastCons (28-way Placental Mammals). The AUC value is given in the lower right corner of each graph.

1. Cluster-Buster


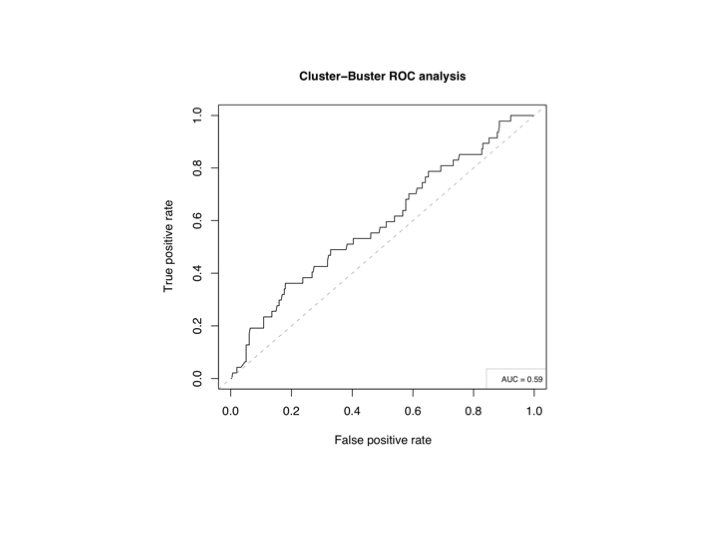


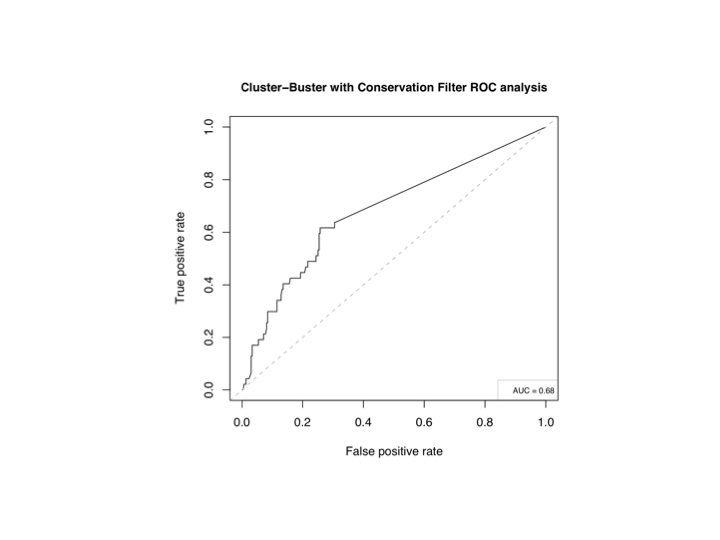


1. LRA
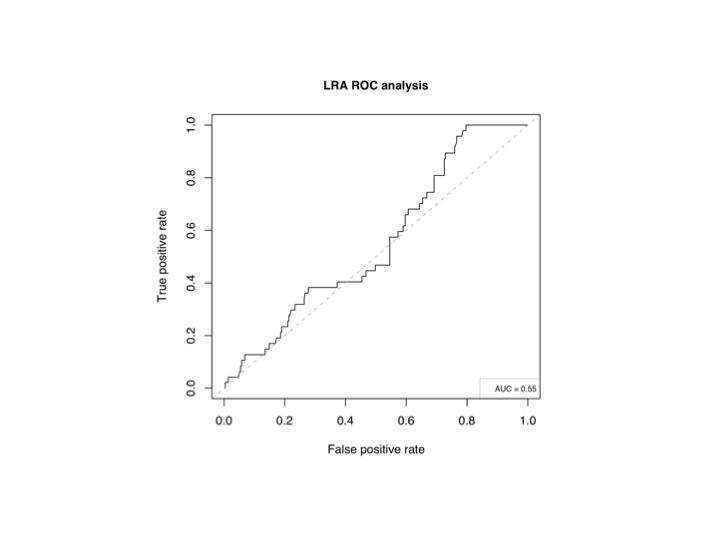
**
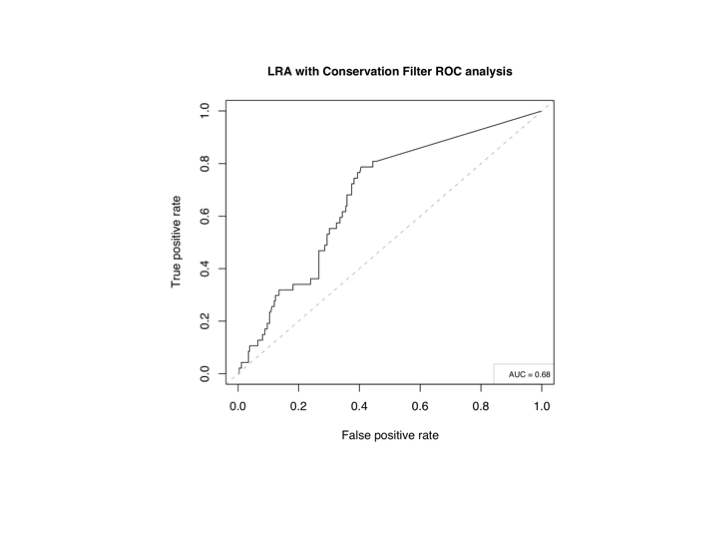
**
2. MSCAN**
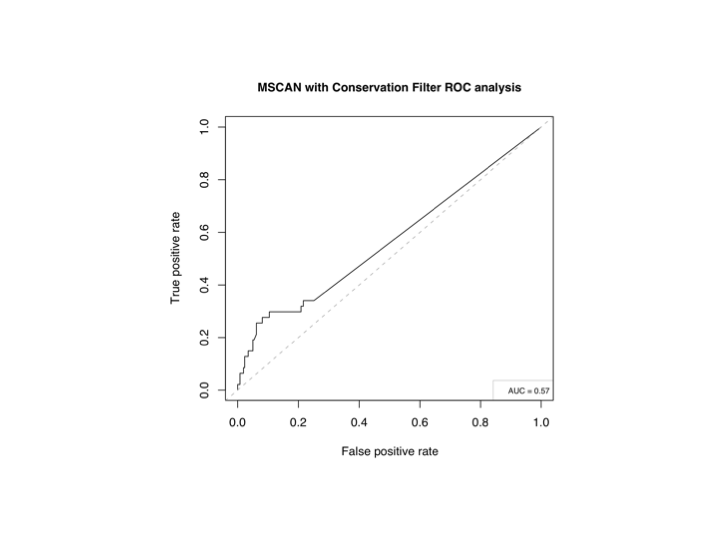

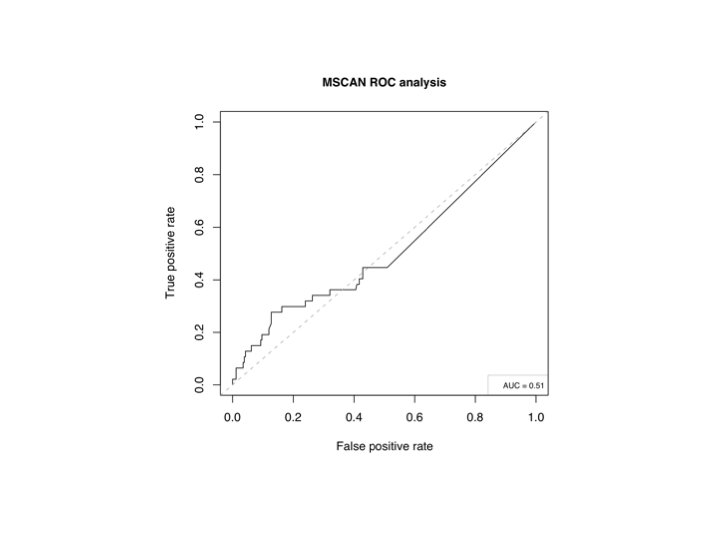
**
3. phastCons


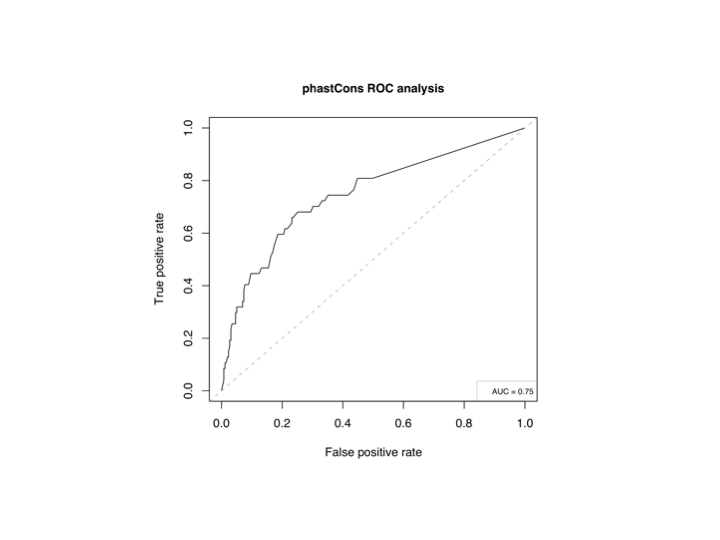


**Supplemental Figure S6.** Density plots for phyloP (46-way All) scores of the predicted binding site positions for the three region sets. The scores for the responding regions (validated and reference) are spread out over a larger range.

**
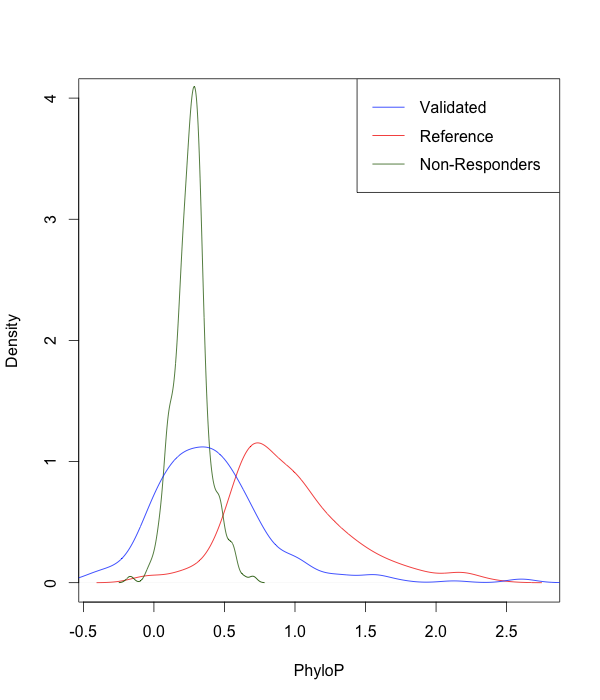
**

**Supplemental Figure S7**. Biases in the location of wells with successful reporter assays.

1. Distribution of validated regions across rows and columns. The number of validated regions in the middle rows and columns tend to be higher compared to others.


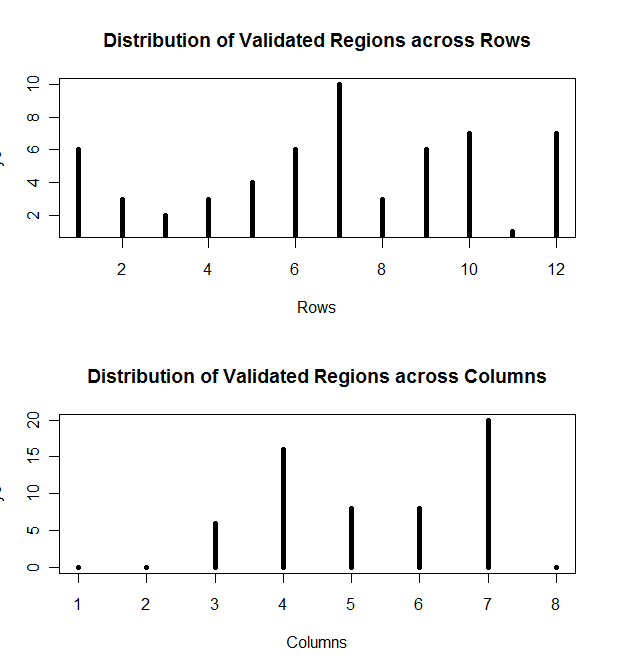


1. Distribution of failed assays across the 96-well plate. The vertical axis represents the count of wells that did not show any significant reporter expression. The 4 corner wells clearly have the highest number of such inactivity.

**
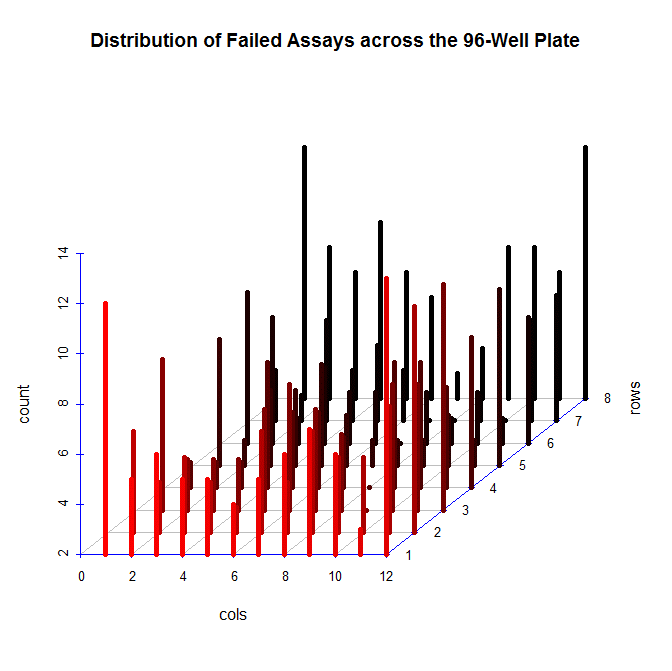
**
