# Supplementary material for: Validation of Skeletal Muscle cis-Regulatory Module Predictions Reveals Nucleotide Composition Bias in Functional Enhancers
Source: PLoS Comput Biol. 2011 Dec 1;7(12):e1002256. doi: 10.1371/journal.pcbi.1002256 (PMC3228787; doi:10.1371/journal.pcbi.1002256)
Supplement: Text S3 — Contains the supplemental notes on technical experimental issues. (DOC) [file pcbi.1002256.s004.doc]

**Technical Notes**

Compared to the initial expectations of the authors, the number of successfully validated muscle-specific CRMs was quite low. One significant contribution to the low success rate was technical failures in the transfection assays. It has been reported that the outer columns and rows in 96-well plates can exhibit lower expression values compared to the inner wells (Malo, Hanley et al. 2006). For this study, no special precautions were taken against such effects because it was expected that any depressed expression would be normalized across the experiments, as each well location was treated independently for analysis. The ultimate measure was the relative expression observed at a position between the myoblasts and myotubes. However, a large fraction of undetectable expression was observed in the outer wells (Supplemental Figure S7 in Text S2).

The data analysis was made more complex by the stochastic nature of the laboratory procedure. Because the plasmids from the clones are picked and transferred randomly to the 96 well plates, the number of replicates for each insert sequence of interest is variable. In this study the replicates range from 1 to 12, but the median was 2.
